# Hierarchical Micro/Nano-Porous Acupuncture Needles Offering Enhanced Therapeutic Properties

**DOI:** 10.1038/srep34061

**Published:** 2016-10-07

**Authors:** Su-ll In, Young S. Gwak, Hye Rim Kim, Abdul Razzaq, Kyeong-Seok Lee, Hee Young Kim, SuChan Chang, Bong Hyo Lee, Craig A. Grimes, Chae Ha Yang

**Affiliations:** 1 Department of Energy Systems Engineering, Daegu Gyeongbuk Institute of Science & Technology (DGIST), 333 Techno Jungang-daero, Hyeonpung-myeon, Dalseong-Gun, Daegu, 42988, Republic of Korea; 2 Department of Physiology, College of Oriental Medicine, Daegu Haany University, 136 Shincheondongro, Suseong-Gu, Daegu, 42158, Republic of Korea; 3 Flux Photon Corporation, 116 Donmoor Court, Garner, North Carolina, 27529, United States

## Abstract

Acupuncture as a therapeutic intervention has been widely used for treatment of many pathophysiological disorders. For achieving improved therapeutic effects, relatively thick acupuncture needles have been frequently used in clinical practice with, in turn, enhanced stimulation intensity. However due to the discomforting nature of the larger-diameter acupuncture needles there is considerable interest in developing advanced acupuncture therapeutical techniques that provide more comfort with improved efficacy. So motivated, we have developed a new class of acupuncture needles, porous acupuncture needles (PANs) with hierarchical micro/nano-scale conical pores upon the surface, fabricated via a simple and well known electrochemical process, with surface area approximately 20 times greater than conventional acupuncture needles. The performance of these high-surface-area PANs is evaluated by monitoring the electrophysiological and behavioral responses from the *in vivo* stimulation of Shenmen (HT7) points in Wistar rats, showing PANs to be more effective in controlling electrophysiological and behavioral responses than conventional acupuncture needles. Comparative analysis of cocaine induced locomotor activity using PANs and thick acupuncture needles shows enhanced performance of PANs with significantly less pain sensation. Our work offers a unique pathway for achieving a comfortable and improved acupuncture therapeutic effect.

Acupuncture has long been accepted as an effective therapy for the treatment of many functional disorders, such as pain and psychiatric disorders including anxiety and drug abuse^1^,^2^,^3^. The invention of acupuncture as a therapeutic treatment is traced as far back as 6000 B.C., originating with the insertion of sharpened stones at specific acupuncture points^1^,^2^. The ancient use of sharp stones as an acupuncture device is replaced by that of fine needles made from various materials including bamboo, ceramic, bone, and plant thorns, with these in turn replaced by metal acupuncture needles, including those of gold, silver, copper, and stainless steel^4^.

The biological basis of acupuncture still remains unclear, however a considerable number of studies has established a general concept that acupuncture contributes to the neurochemical balance in the central nervous system (CNS) and recovery or maintenance of homeostasis^5^,^6^ via interactions between needles and the surrounding tissue^7^,^8^. In our previous study we reported the activation of the A-beta afferent fiber (sensory nerve fiber) of the ulnar nerve promoting cellular activation by acupuncture at Shenmen (HT7) points for modulating cocaine-induced addictive behavior^9^. Moreover, it is found that mechanoreceptors in the superficial and deep afferents of the ulnar nerve play a functional role in producing acupuncture effects during mechanical stimulation of HT7. Involvement of the afferent fibers in acupuncture is supported by studies investigating acupuncture-like stimulation of superficial or deep tissue for reducing micturition contraction of the urinary bladder^10^ and acupuncture analgesia abolished by blockade of afferents fibers from muscle^11^.

In acupuncture therapies manual manipulation of acupuncture needles is still the most practicable clinical procedure to enhance the stimulation intensity for improved therapeutic effects. Various needle parameters such as diameter^12^,^13^, depth of insertion^13^,^14^, number of needles used per session^15^, and needle surface modification^16^,^17^ have been investigated for improved acupuncture performance. These studies suggest that employing thick needles and/or deeper insertion can act to produce increased stimuli intensity. Accordingly, altering the above mentioned parameters can moderately influence the acupuncture efficacy due to improved possible interaction between the inserted needle and mechanoreceptors in the surrounding tissue^7^,^8^. We hypothesize that an increase in needle surface area with no significant variation in needle diameter may lead to increased interactions of surrounding tissue, leading to enhanced acupuncture stimuli. So motivated, we have fabricated a new type of acupuncture needle possessing a novel surface morphology, and applied this as described in the present work. The fabrication methodology employed herein results in so-called porous acupuncture needles (PANs), with hierarchical micro/nano-scale conical pores upon the surface of conventional stainless steel acupuncture needles. The fabricated PANs exhibit approximately 20 times greater surface area than conventional needles. The performance efficacies of both conventional acupuncture needles and PANs are evaluated by electrophysiological and behavioral responses using *in vivo* tests employing Wistar rats. A comparative analysis between PANs and significantly thick acupuncture needles possessing similar surface area, made by measuring cocaine induced locomotor and pain activity, finds superior performance with the PANs. To the best of our knowledge, this is the first report regarding an acupuncture needle possessing an optimally designed surface that provides improved therapeutic effects without loss of comfort.

## Materials and Methods

### Preparation of porous acupuncture needles (PANs)

Conventional stainless steel acupuncture needles, (6 cm length and 0.03 cm diameter, Dong Bang Acupuncture Inc., Republic of Korea) were used. Before anodization the acupuncture needles were sequentially cleaned with acetone, ethanol, and finally rinsed with deionized water (DI). Anodization of the stainless steel needles was performed using a two-electrode cell, with the acupuncture needle serving as the working electrode and carbon paper (Carbon and Fuel cell (CNL), Republic of Korea, 5 cm × 1 cm × 0.042 cm) as the counter electrode. Anodization was carried out for 1 hour (h) with voltage as a variable, using an electrolyte comprised of 0.3 wt% (weight percent) NH_4_F (98%, American Chemical Society (ACS) reagent, Alfa Aesar) and 2.0 vol% (volume percent) DI water in ethylene glycol^18^. After anodization, the acupuncture needles were rinsed with acetone, ethanol, and DI water, and then air-dried in a flowing stream of nitrogen. The schematic view of experimental setup used in preparation of PANs was shown in Supplementary Figure S1.

### Characterization of porous acupuncture needles (PANs)

Needle surfaces were evaluated using a Scanning Electron Microscope (SEM, Hitachi S-4800 High Resolution Scanning Electron Microscope operating at 3 kiloVolt (kV); Energy Dispersive Spectroscopy (EDS, Brucker Co.) operating at 20 kV). UV-visible absorption spectroscopy of the dye solutions was measured using a Cary series UV-visible near IR spectrophotometer (Agilent Technologies) in the range of 550–750 nm.

The electrochemical impedance spectroscopy (EIS) tests were performed using Bio Logics SAS (Model VSP-1158) three-electrode workstation with platinum (Pt) wire as the counter electrode, Ag/AgCl electrode as the reference electrode and acupuncture needles as the working electrode. The system was operated using EC Lab software in the frequency range of 200 kiloHertz (kHz) to 50 MegaHertz (MHz). The electrolyte consists of saline solution (0.9 g NaCl in 100 ml DI water) purchased from JW-Pharma, Republic of Korea and used without further modifications.

### Animal Preparations

The acupuncture efficacy of the PANs was evaluated by *in vivo* monitoring of the electrophysiological and behavioral responses of Wistar rats, and comparing their outcomes to those obtained using conventional acupuncture and relatively thick acupuncture needles with diameter 1.6 mm, so as to possess the same surface area as the PANs. Subjects were male Wistar rats (body weights 250–270 gram (g) at the start of the study) obtained from Orient Bio (Busan, Republic of Korea) and housed with 12 h light/dark cycle and fed ad libitum. Animal experiments were performed in accordance with guideline regulation of the standards of Use Committee and National Institutes of Health (NIH) guidelines and the Institutional Animal Care and Use Committee (IACUC) at the Daegu Haany University. All experimental protocols were approved by the Daegu Haany University IACUC for this specific study (animal use protocol number DHU2014-051) including animal care, housing, auto-controlled housing facility (temperature and humidity) and sanitization. Measurement of behavioral tremor activity and *in vivo* electrophysiology were blindly performed. Post-experimentation; all rats were euthanized by overdose of 100 mg/kg sodium pentobarbital (intraperitoneal injection, i.p.) followed by opening of the thorax.

### Animal models of alcohol dependence

The alcohol group of rats received an ethanol-containing diet, while the paired-fed rats of the control group received the standard liquid diet without ethanol. To establish animal models of alcohol dependence, the rats were given ad libitum access to a commercially available liquid diet (Dyets, Bethlehem, PA, USA) known as the Lieber-Decarli diet^19^. For ethanol self-administration training fresh liquid diets were prepared every few days. To avoid the aversive orosensory properties of ethanol solutions, rats were introduced to ethanol by increasing the percentage of ethanol from 1% to 7% by 7 increments over 7 day periods and then they were maintained at a final concentration of 7.2% for 9 days^20^. Paired-fed rats received the control liquid diet containing maltose dextrin in place of ethanol to ensure an iso-caloric diet compared with an ethanol-containing diet. Paired-fed rats were given the same volume of liquid diet as the ethanol-fed rats consumed the previous day.

### Measurement of neuronal activity

The neuronal response to acupuncture stimulation was investigated in a single spinal dorsal horn neuron using extracellular recording techniques on the stereotoxic frame^21^. Briefly, Wistar rats were anesthetized by sodium pentobarbital (60 mg/kg, i.p.) and a laminectomy of vertebral segments. T12-L1 was performed to expose the lumbar enlargement (lumbar 4–5 regions) known to be innervated by sensory primary afferent fiber input from peripheral receptive fields including the hindpaw. Tracheal and jugular vein cannula were inserted for breathing and infusion of sodium pentobarbital (5 mg/h/300 g), respectively. A constant anesthesia level was maintained during recording of the single dorsal horn neuron activity, recorded using a carbon filament-filled single glass microelectrode (0.4–0.8 MΩ, impedence @ 1 kHz; Kation Scientific, Minneapolis, MN, USA) in the lumbar L4/5 dorsal horn^21^. After identification of single dorsal horn neuron activity, acupuncture stimulation (80 Hertz (Hz), 10 seconds) by a mechanical acupuncture instrument (MAI) was applied to the identified peripheral receptive field at the hindpaw. The single dorsal horn neuron activity was amplified and filtered (ISO80; World Precision Instruments, USA), fed directly into either an oscilloscope (World Precision Instruments, USA) or the data acquisition unit (CED-1401; Cambridge Electronic Design, UK), and stored in the computer to construct the waveforms or peri-stimulus time histograms (spikes/1 second bin width, Spike2 software, http://ced.co.uk/products/spike2). To ensure that recording of neuronal activity in a single dorsal horn was maintained during the experiments action potential shape and amplitude was monitored using Spike2 program (ver 7.02), respectively.

### Acupuncture treatment

Prior to application, the acupuncture needles were cleaned and sterilized. The needles were inserted (3 mm depths, marked by yellow rubber, see Supplementary Figure S2) into the receptive field of the lumbar spinal dorsal horn neuron without bleeding. Only one needle was used for each insertion and stimulation. To deliver constant mechanical stimulation, we used a novel mechanical acupuncture instrument (MAI) producing a consistent vibration stimulation through an alligator clip attached to a vibrator (MB-1203V, Motor bank, Republic of Korea, see Supplementary Figure S2)^9^. Briefly, the tip of the needle was attached to an accelerometer (PO-AXA-12-01, Intellane Co., Republic of Korea) that converted acceleration (of motion) to analog signals during MAI stimulation. The signals from the accelerometer were digitized using a data acquisition card (DAQ-NI USB-6200, National Instruments, USA) and analysis of intensity (acceleration) and frequency of vibration was done by the Labview program (National Instruments, USA). Acupuncture stimulation by 10 seconds using an on/off switch was delivered at the hindpaw receptive field of the spinal dorsal horn neurons in rats.

### Measurement of tremor activity in alcohol-dependent rats

At the end of ethanol self-administration, the ethanol-containing diet was replaced by the control liquid diet for 2 h to precipitate ethanol withdrawal, and then the ethanol withdrawal signs, tremors, were measured^22^. Tremor activity was quantified in a real-time manner using a force-transducer (Grass Instruments, USA) mounted on a restraint holder (20 cm × 6 cm) in an automatic tremor activity monitoring system. The signals from the force-transducer were fed into bridged amplifiers (ETH-200, iWorx CB Sciences, Inc., USA), filtered between 10–22 Hz and quantified using a LabChart & Scope program. We identified ethanol withdrawal tremors by measuring the intensity of motion-power between 10 to 22 Hz at 1 Hz increments, with the average value used for comparison. To ensure the appropriate measurement of tremor activity hamaline, a tremogenic compound, was given (10 mg/kg, s.c.) as a positive control. To test the effects of needle stimulation on tremor activity, acupuncture treatment using conventional needles and PANs were given at bilateral acupoint HT7 (located at the wrist crease on the radial side of the flexor carpiulnaris tendon, between the ulnar and the pisiform bones, respectively). Similar to extracelluar recording experiments, we used MAI for stimulating acupoints. We had previously suggested that acupuncture at HT7 reduced ethanol withdrawal syndrome in Wistar rats^23^.

### Cocaine Induced Locomotor Activity

Locomotor activity was measured using PANs and acupuncture needles of larger diameter (1.6 mm), that possessed the same surface area as the PANs. An individual rat was placed in a square open field black acrylic box (40 cm × 40 cm × 45 cm). The movement of each animal was monitored by a video camera mounted above the box using video tracking software (Ethovision 3.1), with locomotor activity measured using an image analysis system (Ethovision, Noldus Information Technology BV, Wageningen, Netherlands). Before the locomotor activity tests, animals were habituated for 60 min in the open field and baseline activity then recorded for 30 min. The monitored animal was then given an intraperitoneal injection of cocaine (15 mg/kg, i.p.), followed by acupuncture treatment. Locomotor activity data was analyzed over 10 min periods and expressed as a percentage of the recorded baseline activity.

### Pain Sensation Measurement

Further comparison of the different needles was made through measurement of spontaneous pain sensation induced by acupuncture needle insertion using audible Ultrasonic Vocalizations (USVs). Prior to undergoing a USV test a rat was housed in the USV test box (70 cm × 50 cm × 60 cm; with proper air circulation, regulated temperature, and protected from sound and light) for 3 consecutive days to eliminate environmental influences. A high performance microphone (Ultrasonic USB Microphone 250 KHz, Dodotronic, USA) was attached within a hole in the center of the box ceiling 30 cm above the rat. Abisoft-SASLap Pro (Version 5.2, Avisoft Bioacoustics, Glienicke, Germany) was used to record and analyze the USVs during acupuncture treatment. For statistical analysis vocalization waveform shapes in the pain-related USV frequency range (18–35 kHz) were divided into two types: short duration (20–200 ms) and long duration (>200 ms).

### Statistical Analysis

Data was presented using SigmaPlot (Ver. 12). The comparison of electrophysiology and harmaline effects was perfomed using *t-test*, and tremor activity was performed using repeated One-Way ANOVA with host-hoc *Tuekey* test. All significant differences were determined by p < 0.05. Data was expressed by mean ± S.E.

## Results and Discussions

**Figure 1:**
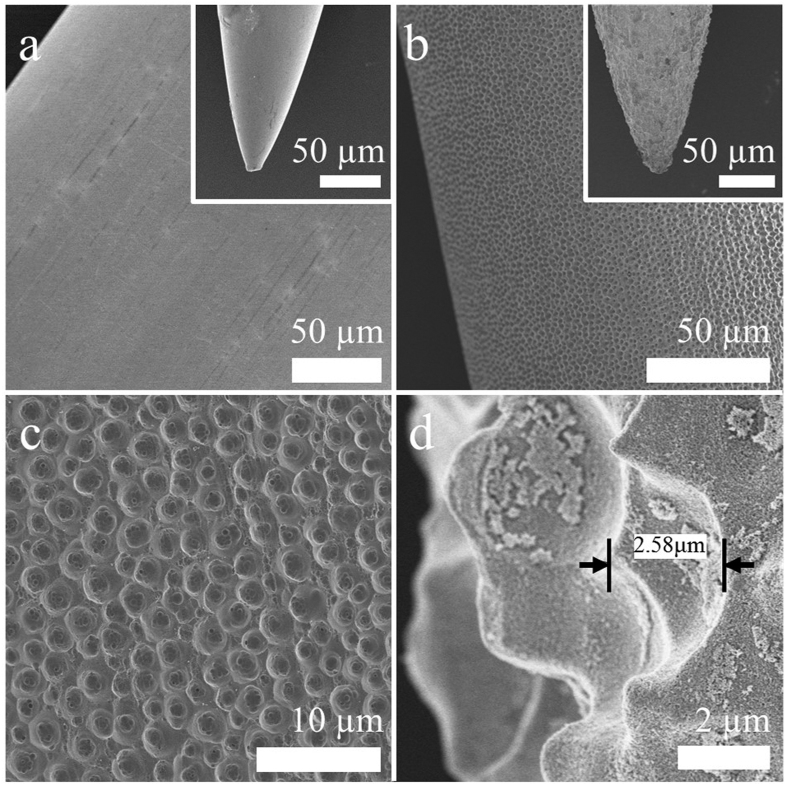
Surface images of: (**a)** Conventional stainless steel needle. (**b)** Porous anodized needle. (**c)** Enlarged image of (**b**). (**d)** Cross-sectional image of the porous anodized needle. Insets of (**a**,**b**) show needle tips.

Figure 1 shows Scanning Electron Microscope images of the conventional and anodized (electrolyte of 0.3 wt% NH_4_F and 2 vol% DI water in ethylene glycol at room temperature for 1 h at variable anodization voltages)^18^ stainless steel acupuncture needle surfaces. It is obvious that the conventional needle possesses a relatively smooth surface, Fig. 1a, while that of the anodized needle has a distinct micro/nano-scale porous topology, Fig. 1b. Uniform micro/nano porous topology is achieved at an anodization voltage of 30 V; see Fig. 1c,d. One can see that the pores are conical in shape, tapering in size from 3.0 μm at the surface to 0.05 μm, with a cone depth varying from 1.0 to 2.6 μm. For the given electrolyte we found an optimal anodization voltage of 30 V, with smaller voltages leading to smaller surface area topologies, and larger voltages resulting in a non-uniform surface topology.

A cross-sectional image of the porous topology is shown in Fig. 1d. Elemental analysis of the acupuncture needle before and after electrochemical anodization, using energy dispersive spectrometer (EDS), is shown in Supplementary Table S1. No formation or loss of any element in the acupuncture needle is detected, assuring that electrochemical anodization doesn’t change the chemistry of the acupuncture needle.

**Figure 2:**
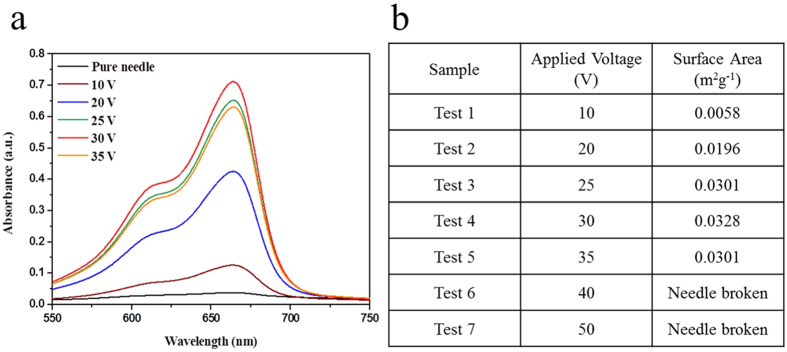
(**a**) The absorption spectra for Methylene Blue dye adsorption shows an enhancement for the 30 V anodized needle. (**b)** Change in needle surface area as a function of anodization voltage, with the 30 V anodized needle showing a maximum result.

Needle surface area is determined using dye desorption measurements in combination with the Beer-Lambert’s Law. The absorption spectra for Methylene Blue dye-adsorbed needles, conventional and anodized, is shown in Fig. 2a. The surface area of our optimal 30 V anodized needle is 0.0328 m^2^g^−1^, which is approximately twenty times greater than that of the starting needle (0.0017 m^2^g^−1^), see Fig. 2b. The calculated needle surface area corresponds to the portion of the needle inserted, or ‘dipped,’ during acupuncture treatment.

**Figure 3:**
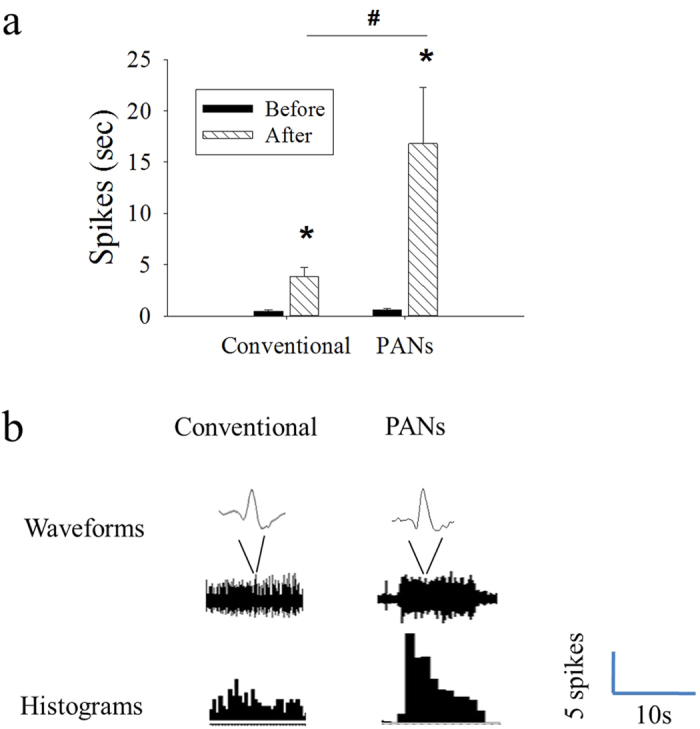
(**a**) Conventional and PAN groups show increased neuronal activity to needle stimulation (after) compared to before stimulation (*p < 0.05). In addition, PAN group shows a significant increase in stimulation-induced neuronal activity compared to conventional group (^#^p < 0.05). (**b)** Real-time waveforms of activity (upper: single action potential shape; lower: waveforms of samples neurons) during *in vivo* spinal single dorsal horn neuron recording, with the resulting histogram of the neuronal activity (bottom row, 1 second bins). The data recording apparatus is illustrated in Supplementary Figure S2.

To determine whether the PANs produce a significantly different electrophysiological response as compared to conventional, smooth-surface needles, the ability of the PANs to facilitate the neural response of a single spinal dorsal horn is tested. Rats receiving acupuncture with conventional needles (n = 6) show spinal dorsal horn neuronal activity (3.85 ± 0.9 spikes/sec) significantly higher than baseline pre-stimulation neuron activity (0.5 ± 0.1 spikes/sec, *p < 0.05; see Fig. 3a). Rats receiving acupuncture with PANs (n = 9) also show significantly enhanced spinal dorsal horn neuronal activity (16.8 ± 5.4 spikes/sec) than baseline pre-stimulation resting activity (0.6 ± 1.1 spikes/sec, *p < 0.05; see Fig. 3a). However, the PAN group also shows a significant enhancement in neuronal activity compared to the conventional group (*p < 0.05). The histogram of response activity and real-time waveforms of single neuronal activity to needle stimulation are shown in Fig. 3b. While the needle insertion itself did not cause a significant change of spontaneous activity, the needle stimulation induced greater activity for the PAN group than conventional group.

**Figure 4:**
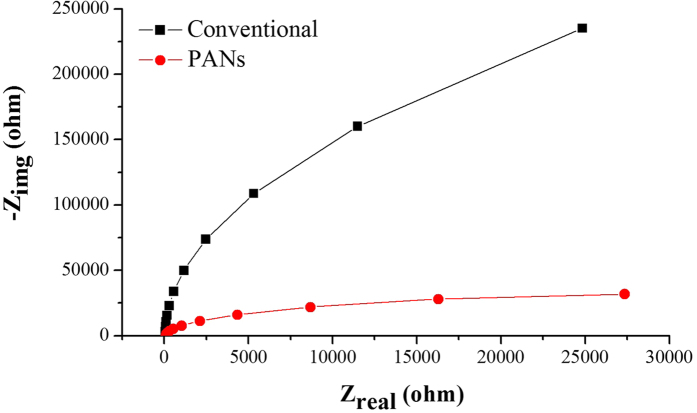
Fitted Nyquist plots corresponding to electrochemical impedance spectra (EIS) for conventional and porous acupuncture needles.

Electrochemical impedance spectroscopy (EIS), a powerful technique for investigating the surface characteristics of materials^24^, is performed for conventional needles and PANs. Figure 4 shows the fitted Nyquist plots corresponding to EIS results for conventional and porous acupuncture needles in saline solution. The Nyquist plots for both needles are of similar shape indicating that the saline electrolyte does not affect the needle surface, while the semicircles of depressed nature can be attributed to the combination of charge transfer resistance (R_ct_) and a constant phase element (CPE) at the working electrode (acupuncture needles in this case) and electrolyte interface^25^. A reasonable decrease can be observed in the semicircle diameter for the PANs, indicating a significant decrease of charge transfer resistance (R_ct_).

**Figure 5:**
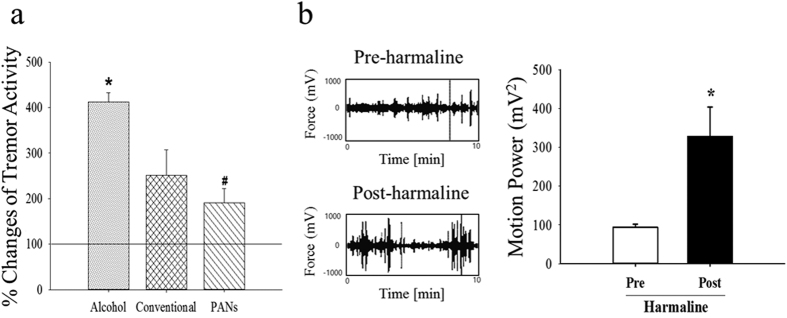
(**a**) Compared to control diet group (Normal as represented by 100%), alcohol treatment group (Alcohol) showed greatly increased tremor activity. PAN group with acupuncture stimulation at HT7 significantly attenuated alcohol-induced tremor activity compared to alcohol group. (**b)** Harmaline, a tremor inducer, was given i.p. to assure proper characterization of tremor activity. Note: In (**a**), *p < 0.05 stands for control and alcohol groups, ^#^p < 0.05 stands for alcohol and PAN groups. In (**b**), *p < 0.05 stands for post- and pre-harmaline groups.

To test the therapeutic effectiveness of the PANs, we analyze the effect of acupuncture using PANs on tremor activity in ethanol-withdrawn rats. The alcohol group (n = 9) demonstrates an elevated tremor activity approximately 4 fold of the control diet group, *p < 0.05, see Fig. 5a. Both the conventional needle group (n = 8) and PAN group have lower tremor activity than the control diet group. However, acupuncture with PANs (n = 10, ^#^p < 0.05) significantly decreases tremor activity compared to that achieved using conventional needles; Fig. 5a. Importantly, the treatment of harmaline significantly increases tremor activity compared with baseline pre-harmaline activity, confirming that tremor activity is correctly measured (Fig. 5b).

**Figure 6:**
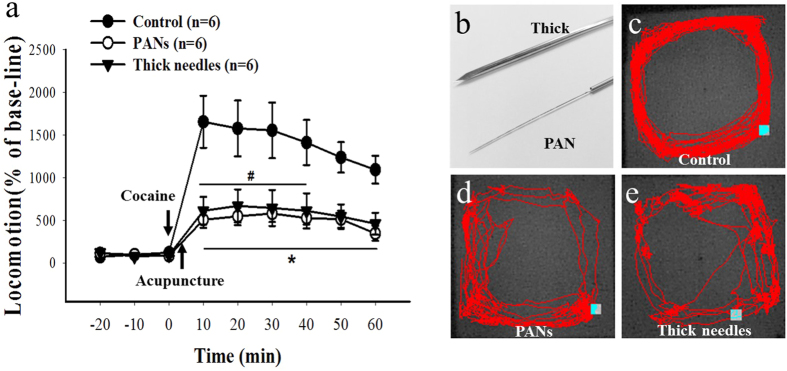
(**a**) Comparison of PANs and thick (1.6 mm diameter) smooth surface acupuncture needles on attenuation of cocaine induced hyper-locomotion (15 mg/kg, i.p.) by HT7 acupuncture. (*p < 0.05 stands for the difference between control and PANs; ^#^p < 0.05 stands for differences between thick acupuncture needle and control. Blue squares in (**c–e**) indicates the start position of the rat).

A comparative analysis between the PANs (0.3 mm diameter) and thick (1.6 mm diameter) acupuncture needles is done using cocaine induced locomotor activity and pain sensation measurements, with all needles inserted into the animal to a depth of 3 mm. Figure 6a shows the attenuation effect of PANs and thick acupuncture needles for the hyper-locomotion induced by cocaine administration (15 mg/kg. i.p.). As compared to the cocaine group (control group), HT7 acupuncture using PANs (*p < 0.05) and thick acupuncture needles (^#^p < 0.05) shows a significantly attenuated hyper-locomotor activity. However over extended duration the PAN group shows a moderate attenuation effect as compared to the thick acupuncture needles. Figure 6b shows pictures of the PANs and thick acupuncture needles selected for comparison studies. Figure 6c–e display the video camera recording of locomotion track activity. It is obvious that PAN (Fig. 6d) and thick acupuncture needle groups (Fig. 6e) show less locomotion activity with similar pattern as compared to cocaine group (control group).

**Figure 7:**
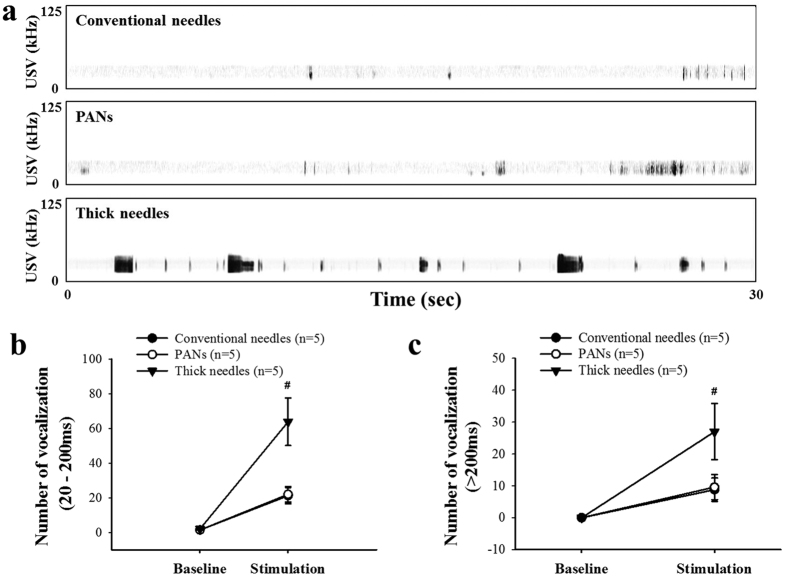
Spontaneous pain sensation measurements for acupuncture needle insertions. (**a**) The typical waveforms of vocalization for conventional needles (0.3 mm diameter), PANs (0.3 mm diameter), and thick (1.6 mm diameter) acupuncture needles, respectively. (**b**) Insertion of the thick acupuncture needles results in significantly increased USVs of both short (20–200 ms) and long (>200 ms) duration.

The pain sensation caused by acupuncture related insertion of conventional, PANs, and thick acupuncture needles is analyzed via audible Ultrasonic Vocalization (USV) tests. USV is considered a reliable tool for detection of spontaneous pain in animal studies; specifically, pain-related vocalizations in animals are found within the frequency range of 18–35 kHz^26^. Typical USVs waveforms measured upon insertion of conventional (smooth surface, 0.3 mm diameter), PAN, and thick needle groups are shown in Fig. 7a. It is clearly seen that the thick needles produce more waveforms as compared to PANs and conventional needles, indicating more pain. Further, thick acupuncture needles show significantly increased USVs over both short, i.e. 20–200 ms (Fig. 7b) and long i.e. >200 ms (Fig. 7c) durations.

The ability of acupuncture to modulate cellular signaling pathways in various pathophysiological conditions is well documented^27^. In the present study, changes of neuronal activity induced by acupuncture needle stimulation are measured as a function of needle-type. In comparison to conventional smooth-surface stainless steel needles of the same diameter, the high surface area PANs demonstrate a significantly greater effect on electrophysiological and behavioral responses. Both PANs and the thick acupuncture needles of the same interfacial surface area show attenuation of cocaine induced hyper-locomotor activity, with the PANs showing significantly reduced pain sensation.

Based on our previous findings, HT7 acupuncture activates A-beta afferent fibers of the ulnar nerves^9^; we find PANs to be more effective than conventional needles, a result that can be associated to increased needle surface area leading to increased interaction of surrounding tissue with acupuncture stimuli. The result indicates that acupuncture with PANs can activate mechanoreceptors more effectively than conventional needles. Our earlier study found that acupuncture at HT7 can suppress the reduction of mesolimbic dopamine neurotransmission^28^ and behavioral withdrawal signs including tremor in ethanol-withdrawn rats^23^; herein, as compared to conventional and thick acupuncture needles we find the PANs have a strong inhibitory effect on ethanol withdrawal tremors as well as cocaine locomotion.

## Conclusions

In conclusion, the electrophysiological and behavioral data demonstrate the higher efficacy of PANs in psychiatric treatment over conventional needles. Further, a comparative analysis of pain sensation measurements using PANs and thick acupuncture needles of the same interfacial surface area clearly shows reduced pain sensation levels with the PANs. From the present study we suggest the possibility of PANs for the advanced *in vivo* treatment of neuropsychiatric disorders. Our important results suggest the motivation for future studies, such as whether the increased needle surface area enhances the conductance of neuronal impulses or initiates specific biochemical events that result in the improved therapeutic efficacy of psychiatric disorders. We believe our present results employing PANs provide researchers new insights that should help improve acupuncture efficacy.

## Additional Information

**How to cite this article**: In, S.-I. *et al*. Hierarchical Micro/Nano-Porous Acupuncture Needles Offering Enhanced Therapeutic Properties. *Sci. Rep.* **6**, 34061; doi: 10.1038/srep34061 (2016).

## Supplementary Material

Supplementary Information
