# Supplementary material for: Hierarchical Micro/Nano-Porous Acupuncture Needles Offering Enhanced Therapeutic Properties
Source: Sci Rep. 2016 Oct 7;6:34061. doi: 10.1038/srep34061 (PMC5054419; doi:10.1038/srep34061)
Supplement: Supplementary Information [file srep34061-s1.doc]

Hierarchical Micro/Nano-Porous Acupuncture Needles Offering Enhanced Therapeutic Properties

Su-Il In^a,†,*^, Young S. Gwak^b,†^, Hye Rim Kim^a^, Abdul Razzaq^a^, Kyeong-Seok Lee^a^, Hee Young Kim^b^, SuChan Chang^b^, Bong Hyo Lee^b^, Craig A. Grimes^c^, and Chae Ha Yang^b,*^

[^a^]Department of Energy Systems Engineering, Daegu Gyeongbuk Institute of Science & Technology (DGIST), 333 Techno Jungang-daero, Hyeonpung-myeon, Dalseong-Gun, Daegu, 42988, Republic of Korea.

[^b^] Department of Physiology, College of Oriental Medicine, Daegu Haany University, 136 Shincheondongro, Suseong-Gu, Daegu, 42158, Republic of Korea.

[^c^] Flux Photon Corporation, 116 Donmoor Court, Garner, North Carolina, 27529, United States.

[^†^] These authors contributed equally to this work.

[^*^] Coresponding authors ([insuil@dgist.ac.kr](mailto:insuil@dgist.ac.kr) and [chyang@dhu.ac.kr](mailto:chyang@dhu.ac.kr))


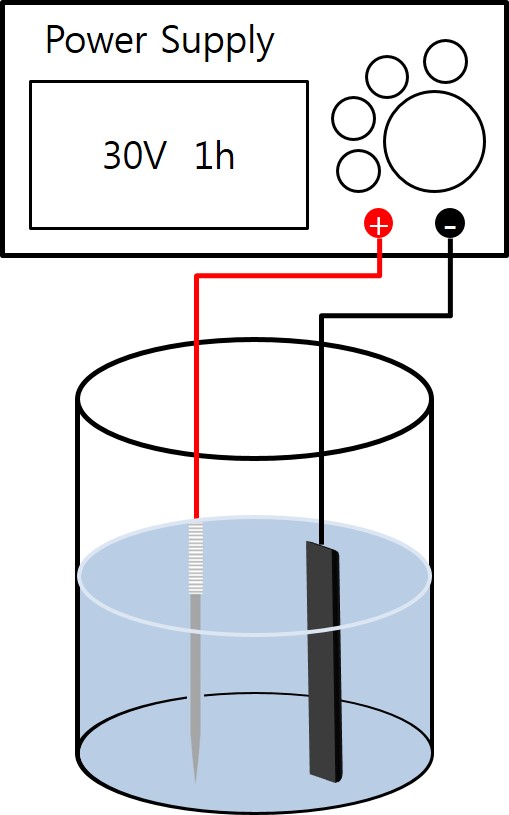


**Figure S1.** Schematic diagram showing the anodization setup used in this research.


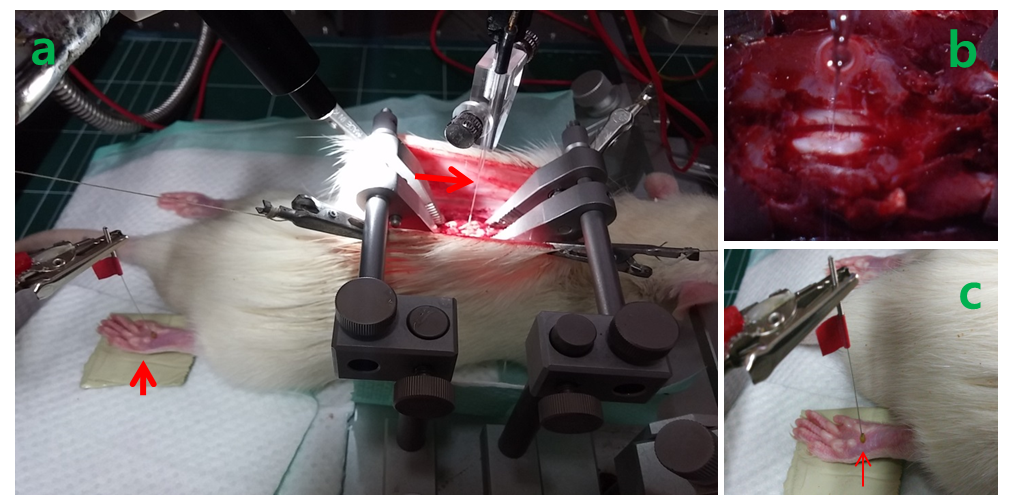


**Figure S2.** **(a)** The overview of *in vivo* electrophysiology. After anesthesia, a lumbar laminectomy exposed the lumbar spinal cord and framed on the stereotoxic frame. **(b)** To record the neuronal response activity, the carbon-glass electrode (arrow, red) was inserted into the lumbar 4/5 spinal dorsal horn by micropositioner. **(c)** The acupuncture stimulation was induced by automatic mechanical acupuncture instrument (MAI) designed to produce the consistent vibration stimulation through an alligator clip attached to a vibrator. The red arrow head indicates the insertion of acupuncture needle at the hindpaw, a receptive field of lumbar 4/5 spinal dorsal horn neurons, and the yellow square rubber (attached at the needle, red thin arrow) adjust the consistent insertion (3 mm into the skin) during the acupuncture stimulation.

**Table S1. EDS (Energy Dispersive Spectrometer) data before and after anodization of needles**

|  | **Before Anodization** | | **After Anodization** | |
| --- | --- | --- | --- | --- |
| **Elements** | **Atom. C (at. %)** | **Error (%)** | **Atom. C (at. %)** | **Error (%)** |
| Fe | 44.76 | 1.76 | 45.47 | 1.28 |
| Cr | 12.19 | 0.50 | 18.29 | 0.55 |
| C | 36.29 | 2.73 | 31.75 | 2.31 |
| Ni | 5.69 | 0.34 | 2.76 | 0.18 |
| Al | 1.08 | 0.09 | 1.73 | 0.12 |
